# Wearable Sensors and Systems for Wound Healing-Related pH and Temperature Detection

**DOI:** 10.3390/mi12040430

**Published:** 2021-04-14

**Authors:** Ning Tang, Youbin Zheng, Xue Jiang, Cheng Zhou, Han Jin, Ke Jin, Weiwei Wu, Hossam Haick

**Affiliations:** 1 School of Aerospace Science and Technology, Xidian University, Xi’an 710126, China; ntang@tju.edu.cn; 2 Department of Chemical Engineering and Russell Berrie Nanotechnology Institute, Technion-Israel Institute of Technology, Haifa 3200003, Israel; youbinzheng@campus.technion.ac.il; 3 School of Advanced Materials and Nanotechnology, Xidian University, Xi’an 710126, China; jiangx2020@stu.xidian.edu.cn (X.J.); wwwu@xidian.edu.cn (W.W.); 4 Institute of Micro-Nano Science and Technology, Shanghai Jiao Tong University, Shanghai 200240, China; chengzhou@tju.edu.cn

**Keywords:** wearable sensor, smart systems, pH, temperature, wound management

## Abstract

Wound healing is a complex tissue regeneration process involving many changes in multiple physiological parameters. The pH and temperature of a wound site have long been recognized as important biomarkers for assessing wound healing status. For effective wound management, wound dressings integrated with wearable sensors and systems used for continuous monitoring of pH and temperature have received much attention in recent years. Herein, recent advances in the development of wearable pH and temperature sensors and systems based on different sensing mechanisms for wound status monitoring and treatment are comprehensively summarized. Challenges in the areas of sensing performance, infection identification threshold, large-area 3-dimensional detection, and long-term reliable monitoring in current wearable sensors/systems and emerging solutions are emphasized, providing critical insights into the development of wearable sensors and systems for wound healing monitoring and management.

## 1. Introduction

Our skin is important in protecting internal organs from external environmental hazards [1]. However, in some cases, it may be subject to injuries, such as cuts, abrasions, blisters, burns, stab wounds, etc. Generally, current treatment of skin wounds is simply to place a bandage or medical gauze to prevent the wounds from being infected by infectious microorganisms (e.g., pathogens) [2,3,4]. In clinical practice, the assessment and monitoring of wound status are usually based on visual evaluation of variables (e.g., the color of the wound bed, the amount of exudate) [5]. However, this subjective observation can lead to inconsistencies due to changes in illumination and angles. Meanwhile, these assessments require frequent removal of wound dressing, which will not only waste clinical resources but also interrupt the normal wound healing process, causing a second injury to the wound and pain to the patients [6,7,8]. Therefore, providing some useful real-time information related to the state of wound healing to eliminate the time lag and avoid the formation of chronic wounds is crucial for clinicians to conduct clinical care and make appropriate treatment decisions.

The wound healing process is complex and multi-stage, and different stages are often accompanied by different changes in the physiological environment of the wound, such as the increase of the local temperature, pH alkalization, volatile organic compounds (VOCs) and the abnormal release amount of metabolite, etc. [9,10,11,12,13]. Meanwhile, growth factors, bacteria, and some metabolic byproducts, etc., can also be effective indicators of assessing the degree of wound healing. Thus, these diverse physiological changes, especially pH and temperature, can be considered as biomarkers that provide important physiological information during wound healing (e.g., vascularization and inflammation). Based on the properties, these biomarkers can be roughly divided into biochemical markers (e.g., pH, uric acid) and physical markers (e.g., temperature, pressure) [5,14,15,16,17,18]. Therefore, detection of the changes of these markers provides an effective way for real-time monitoring of wound status.

**Figure 1:**
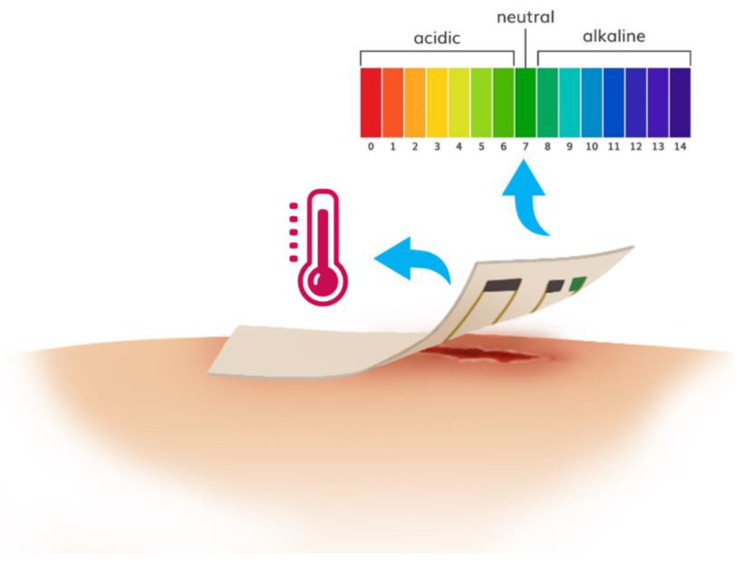
Wearable sensors/systems for wound related biomarkers (e.g., pH, temperature) detection to monitor wound healing status.

Wearable sensors and systems refer to the devices that can deal with trace biological or chemical analytes and convert the chemical reactions or physical changes into usable signals (optical and/or electrical signals, etc.) according to certain rules [19,20,21]. Wearable sensors/systems used for monitoring markers in or around wound environment can provide real-time symptom information and offer promise in therapy studies [22], which also meets the requirements of the World Union of Wound Healing Societies that “diagnostic tools need to be moved into the clinic or the patient’s home to ensure optimal care is provided for patients with wound” [23,24,25]. Researchers have developed many different wearable sensors/systems based on optical (fluorescence, colorimetry, etc.) and/or electrical (impedance, potentiometry, amperometry, etc.) mechanisms, which can integrate with conventional wound dressing to form smart wound dressing to convert the changes of these biomarkers into visual and/or electrical signals to achieve real-time monitoring of wound healing status (Figure 1). There are some good reviews related to smart wound dressing but they do not include more recent wound dressing development based on wound biomarker detection [9,22,26]. The aim of this review is to provide an overview of different researches that have been conducted in the monitoring of two widely used wound biomarkers (pH and temperature) by wearable sensors through different sensing mechanisms.

## 2. Wearable pH Sensors and Systems for Wound Monitoring

**Figure 2:**
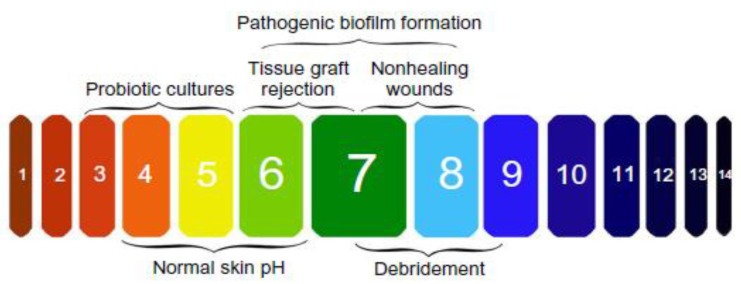
Changes in pH during different wound status and importance of managing pH in chronic wound. (Reprinted from [33]).

The pH within wound milieu is a significant biochemical marker, which has been proved to be an important contributing factor throughout the wound healing process [9,27]. In general, the pH of normal skin and healing wounds is between 4–6.5 (i.e., acidic) [28,29], which is optimal for promoting angiogenesis and epithelization, assisting the release of oxygen and maintaining resident commensal bacteria [30,31,32]. However, in infected wounds, the pH becomes alkaline (above 6.5) which have been linked predominately to the presence of bacteria (Figure 2) [14,33]. Thus, the pH has been identified as an essential diagnostic parameter to follow possible infection. With the increasing interest in pH analysis within wound milieu, pH sensing platforms based on different optical [34,35,36,37,38,39,40,41,42,43] or electrochemical [28,44,45,46,47,48,49] methods have been developed for in-situ and real-time monitoring and analysis of wound healing status.

### 2.1. Wearable Optical pH Sensors and Systems

**Figure 3:**
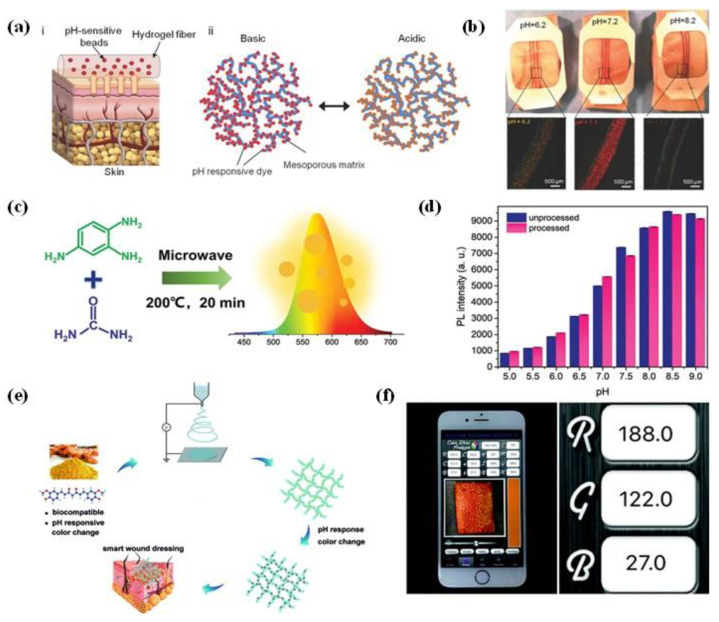
(**a**) Schematic illustration of alginate-based hydrogel microfibers loaded with pH-responsive beads (i) and the action mechanism of solid matrix of the mesoporous particles (ii); (**b**) the wound dressings fabricated by hydrogel microfibers response to different pH (Reprinted with permission from [51]); (**c**) synthesis of orange-emissive carbon quantum dots (O-CDs); (**d**) comparison of photoluminescence intensities of O-CDs on medical cotton cloth (MCC) before and after immersion in phosphate-buffered saline (PBS) buffers with different pH (Reprinted with permission from [52]); (**e**) fabrication process of curcumin-loaded fibers used for wound pH detection; (**f**) RGB (red, green, blue) values of polymer fibers after pH detection were determined by smartphone (Reprinted with permission from [36]).

Optical pH sensors/systems contain indicator dyes, which show color changes depending on the pH, can be used to monitor pH variation [50]. These sensors can be used for pH recognition without integrating electronics. However, a key challenge for this type of sensor is to ensure tight adhesion between the dyes and wound dressing to prevent the dye from leaching out into the wound. In one attempt to address this challenge, alginate-based hydrogel microfibers embedded with mesoporous particles functionalized with pH-sensitive dyes was fabricated through the microfluidic spinning method of Khademhosseini et al. (Figure 3a) [51]. The pH dyes can be stably combined with the mesoporous matrix through electrostatic interaction, thereby preventing dye leakage from fibers to the wound area. These pH-responsive microfibers were then assembled on a transparent medical tape to create a wound dressing for the long-term monitoring of wound within the range of pH 5.5–9 (Figure 3b). Similarly, Yang et al., have synthesized an orange-emissive carbon quantum dots (O-CDs) that can exhibit pH-indicating color change from red to yellow as the pH value increases from 5.0 to 9.0 [52]. The O-CDs with excitation-independent photoluminescence performance were readily designed through microwave-assisted heating of urea and 1,2,4-triaminabenzene solution at 200 °C (Figure 3c). In this case, the surface of nanosized O-CDs has many different functional groups (i.e., hydroxyl, amino, and carboxyl). Therefore, strong hydrogen bonding interaction can be formed between O-CDs and medical cotton cloth (MCC), which makes it difficult for O-CDs to peel off from cotton fibers, resulting in a good resistance leachability of O-CDs-based wound dressing (Figure 3d). Furthermore, the use of O-CDs-based wound dressing is not affected by blood contamination and long-term storage during pH detection, which provides a reliable strategy for wound status monitoring via visual determination.

In addition to microfluidic spinning, electrospinning technique was also developed to produce pH-responsive fibers with improved sensitivity for wound monitoring [53,54]. Recently, Pan et al., have developed a curcumin-loaded polycaprolactone (PCL) fibrous mat through electrospinning with the capable of in-situ real-time monitoring the wound pH (Figure 3e) [36]. Due to excellent flexibility, PCL/curcumin fibrous mat can be processed into different shapes for different needs. Specifically, the mat could not only be cut into different 2D shapes (e.g., round, rectangular), but can also be easily prepared to a 3D tube structure with controllable diameters, which effectively broaden their usage in wound treatment. During pH monitoring, the composite PCL/curcumin dressing underwent apparent and visible color changes when exposed to pH from 6.0 to 9.0. The RGB values of each color picture of composite dressing after pH detection can be extracted with a portable smartphone and could then be used to establish the relationship with the pH values (Figure 3f). In this way, this kind of wearable sensors could provide a convenient and comfortable approach for patients to reduce the length in hospital and accelerate wound healing. Although optical pH-responsive wearable sensors are easy to miniaturize and not subject to electromagnetic interference, they still face a major disadvantage, that is, they are easily affected by external light conditions, which make it difficult to accurately determine the pH values of the wound.

### 2.2. Wearable Electrochemical pH Sensors and Systems

For more precise detection, electrochemical approaches that have innate selectivity and sensitivity towards target analytes were applied to transduce wound biomarkers concentration into potential, current or/and impedance. In terms of the flexible electrochemical sensors used for healthcare applications, organic and polymer materials have been widely employed as sensing element for pH detection [55,56]. Polyaniline (PANI, one of the commonly used conducting polymers) has shown the intrinsic antibacterial and biocompatible properties [57].

**Figure 4:**
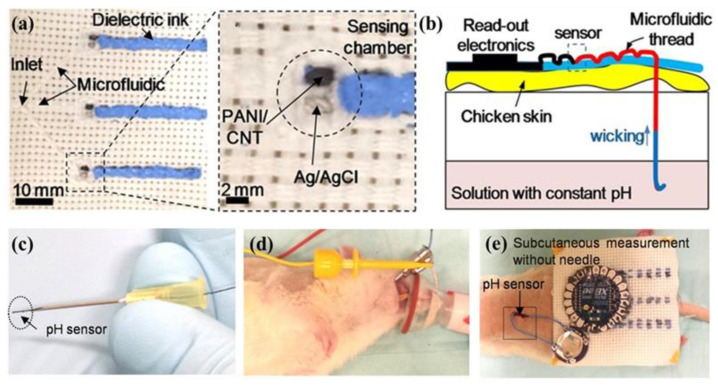
(**a**) Optical images of conductive thread-based pH sensor array; (**b**) mechanism of microfluidic pH sensor in skin wound pH detection; (**c**) the thread-based pH sensor passed through a normal needle; images of the thread-based pH sensor used for (**d**) stomach and (**e**) subcutaneous pH sensing (Reprinted from [58]).

Pooria et al., have developed a conductive thread-based pH sensor with a microfluidic splitter which was used to deliver the sample to the sensing chambers (Figure 4a) [58]. In this system, the carbon nanotube (CNT) coated with doped PANI was dipped on the cotton threads and applied as the working electrode to measure the open-circuit potential with respect to a Ag/AgCl reference electrode. As shown in Figure 4b, the hydrophilic thread was passed through a chicken skin to deliver the solution from a reservoir to mimic subcutaneous pH measurements. The wicking property of the hydrophilic thread allows the samples to diffuse along the suture without significant leakage due to the presence of hydrophobic layer on the chicken skin. Thereafter, the recorded data from thread-based pH sensors were sent wirelessly through the read-out electronics. Because of its small size and flexibility, the thread-based pH sensor can easily pass through a normal needle (Figure 4c), and can be directly inserted into the stomach or implanted under the skin for in vivo measurements (Figure 4d,e). Based on these properties of the thread-based pH sensor, the same group has designed a pH-mapping smart-bandage for chronic wound monitoring [59]. Seven pH sensors were integrated into a commercial bandage through a standard sewing stitch. With an Arduino Nano and Bluetooth module, the wireless data can be sent to the computer and mapped to the corresponding pH values in different areas on the bandage. However, this stitch pH-mapping sensor array had a low resolution for a deep wound sensing. To mitigate this issue, Lyu et al., have reported a new pH sensor array with high surface area electrodes consisting of 3D sewn PANI structure to improve the sensing ability of smart bandage for monitoring deep and non-uniform wounds [60]. These results demonstrated that the thread-based pH sensors have the potential to integrate with textile or fabric and compose smart bandages to monitor wound healing or point-of-care diagnostics.

Comparison of sensitivity/accuracy of wearable pH sensors based on different sensing mechanisms are summarized in Table 1. In addition to the above developments, there are some other pH sensors based on different sensing materials (e.g., carbon/PANI [61], MWCNT/PANI [48], graphite [45], poly-L-tryptophan [44], and CuO nanorod [62]) that have been considered to be used in monitoring wound pH. Regardless of the above methods or sensing materials, recent advances in pH detection have shown that pH is an important biochemical marker for real-time monitoring of the wound status.

## 3. Wearable Temperature Sensors and Systems for Wound Monitoring

Temperature variation has been recognized as an important parameter associated with wound inflammation and infection, since a series of chemical and enzymatic actions involved in the wound healing process are affected by abnormal temperature changes [64,65,66,67]. On one hand, the temperature of the acute wound increased by local vascular expansion, thereby transporting more oxygen and nutrients to the injured site. The temperature of spontaneously healed wounds is higher than 37.8 °C, but not significantly higher than the temperature around the wound. A sudden increase in temperature in the chronic wound area is a sign of infection [67,68,69]. On the other hand, a decrease of the local temperature indicates that the wound is likely to suffer local ischemia, which is also a threat to wound rehabilitation [69,70,71]. Studies have shown that a 2.2 °C variation in temperature could be a warning threshold for impending wound deterioration [72,73]. Therefore, temperature monitoring has great potential as an effective method of assessing wound status [74]. There are several sensors used to measure temperature based on different detection mechanisms, such as infrared sensors, thermistors, and resistance temperature sensors.

### 3.1. Wearable Optical Temperature Sensors and Systems

Nowadays, infrared sensors-based portable devices that provide objective thermal images for clinical tissue structural monitoring are becoming increasingly important in wound assessment [75,76,77]. Forward looking infrared (FLIR) camera systems integrated with smartphone or iPad were widely used in the thermal imaging technology filed to produce thermal images with high 2 dimensions (2D) resolution, improving the efficiency of diagnosis and treatment of wound lesions [78,79,80]. However, despite the advantage for home-monitoring applications, the key limitation of infrared sensors-based thermal imaging technology is that the wound dressing has to be removed during the detection, which might cause a second injury to the wound [81].

**Figure 5:**
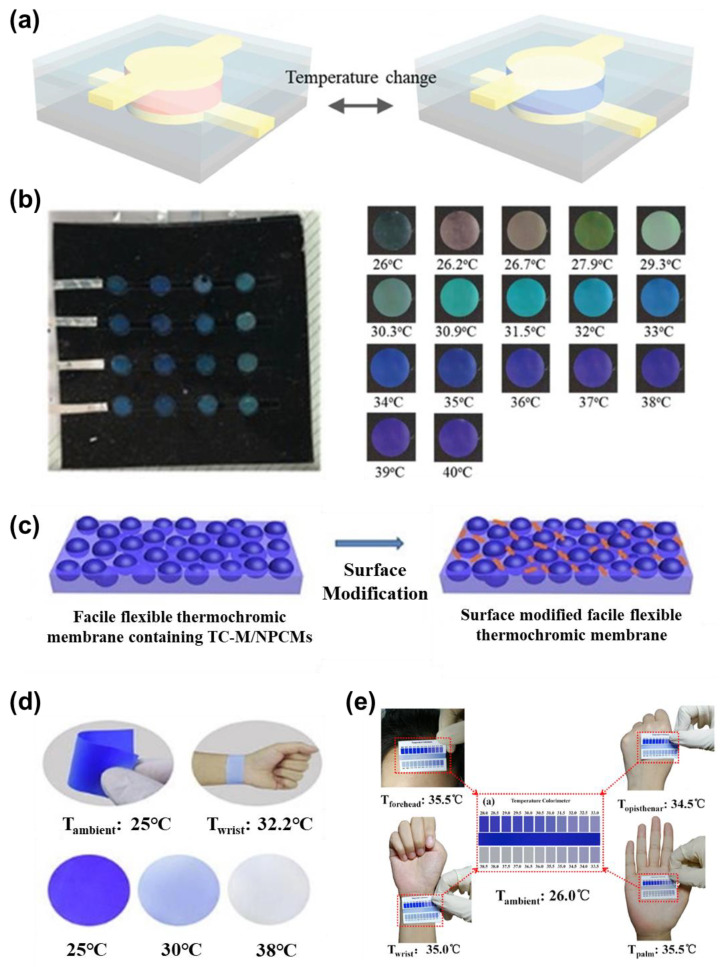
(**a**) Schematic illustration of colorimetric responsibility of optical temperature sensor based on thermochromic liquid crystal (CTL); (**b**) optical image of the sensing unit of wearable temperature sensor at different temperatures (Reprinted with permission from [85]); (**c**) the fabrication process of thermochromic membrane containing TC-M/NPCMs; (**d**) colorimetric response and application of the colorimetric temperature sensor; (**e**) applications of temperature colorimeter in different positions of body surface. (Reprinted with permission from [86]).

In order to make up for the above defects in traditional infrared sensors, wearable sensors with the ability of temperature visualization offers a promising approach with in-situ monitoring of temperature change and real-time diagnosis of the health status by seamless contact with human skin [82]. Thermochromic materials that change color with temperature changes have been widely studied and used in human healthcare [83,84]. Hemant et al., introduced a bimodal sensor array which was fabricated by a feasible method to detect the changes in temperature and pressure in a visible way [85]. As shown in Figure 5a, the elastic composite material containing thermochromic dyes is sandwiched by the stretchable electrode of an amphoteric electrolyte hydrogel to realize the bimodal functions of thermochromic and tactile sensing. Colorimetric responses to different temperatures are shown in Figure 5b, which indicates the good sensitivity of the elastic composite material. In order to quantify the performance of the material, digital image processing was used by identifying particular temperatures based on the hue (in the hue saturation value space of Figure 5b). From the temperature-hue relationship, the temperature can be quantified with an uncertainty value of ~0.1 °C between 26 and 34 °C, and uncertainty was deteriorated close to 0.4 °C near 40 °C. To optimize the accuracy of the colorimetric temperature detection, He et al. fabricated a reversible thermochromic membrane by burying thermochromic microcapsules inside the polyvinyl alcohol/polyurethane (Figure 5c) [86]. The thermochromic membrane exhibited a dual functional feature of effective temperature adjustment and reversible thermal indication (Figure 5d,e). Therefore, this type of membrane has great potential as a wearable temperature for human healthcare monitoring.

### 3.2. Wearable Electrical Temperature Sensors and Systems

With the development of wearable electronics, multifunctional wearable devices are widely used in healthcare and intelligent monitoring [87]. To continuously monitor the wound and/or periwound temperature, wearable flexible temperature sensors have strong practicability compared to other measuring devices, especially for real-time monitoring of the wound status, which is inseparable from accurate temperature detection [88]. Due to low-cost fabrication techniques and low power consumption, wearable resistive temperature sensors have attracted wide interest in integrating into electronic devices for healthcare monitoring.

**Figure 6:**
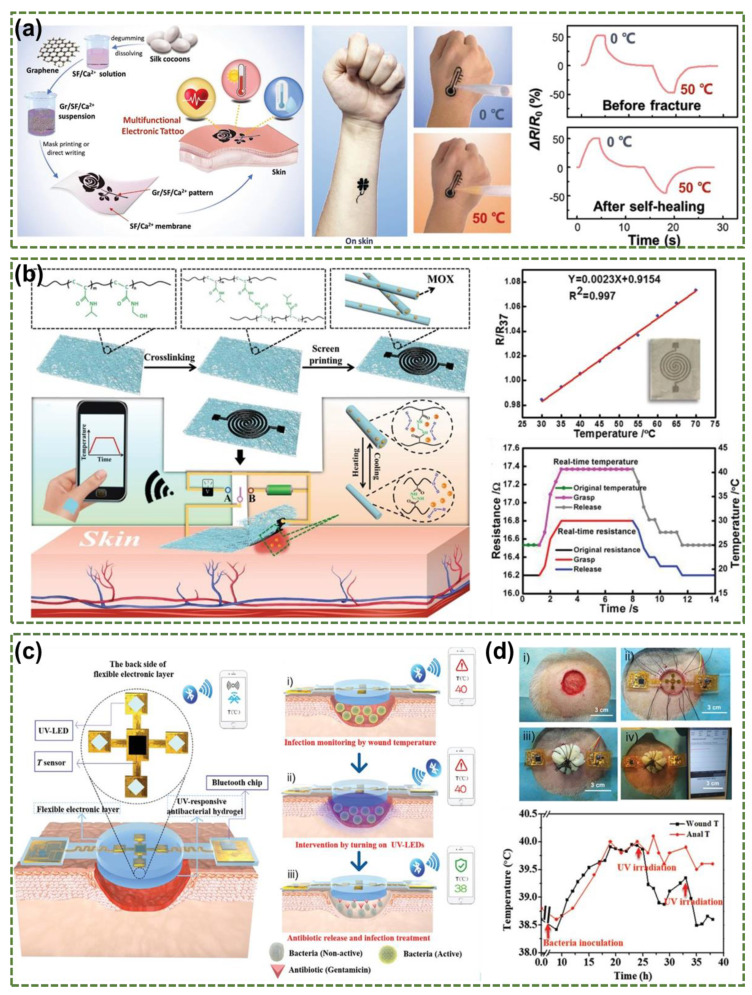
(**a**) Schematic illustration of the fabrication process of Gr/SF/Ca^2+^ E-tattoo and relative resistance changes of pristine and healed temperature sensors (Reprinted with permission from [89]). (**b**) The fabrication and temperature sensing performance of flexible and breathable on-skin devices featuring temperature triggered drug release ability (Reprinted with permission from [99]). (**c**) Schematic illustration of the structures and working principles of the smart wound dressing integrated with flexible electronics and UV-triggered drug release: (i) real-time monitoring of wound temperature; (ii) turning on UV light to trigger the drug release; (iii) infection inhibition, resulting in a decreased wound temperature; (**d**) In vivo studies of the smart wound dressing on infection monitoring and timely treatment: (i) wound creation; (ii) implantation of smart system; (iii) pressure dressing; (iv) real-time temperature monitoring. (Reprinted from [101]).

Wang et al., have described a temperature-responsive self-healable electronic tattoos (E-tattoos) based on a graphene/silk fibroin/Ga^2+^ (Gr/SF/Ca^2+^) that could be transferred onto the skin [89]. The fabrication processes and applications of the multifunctional E-tattoos are shown in Figure 6a. Due to the variation of electron hopping at the interface between adjacent graphene sheets, the resistance of the E-tattoos changed with different temperature. We notice that the resistances were changed by loading and removing of the water with different temperatures, which demonstrates the ability of the E-tattoos to perceive the external temperature. Self-healing is used to improve the durability and life-time of the sensors and materials [90,91,92,93,94]. The E-tattoos have great healing efficiency and can 100% healed in just 0.3 s, and there are no significant changes in the sensing performance before and after self-healing. To improve the response speed, Zhou et al., proposed a new strategy to improve the thermal sensing performance of PEDOT: PSS by combining micro/nano confinement and materials doping through fabricating different sized micro/nanowires [95]. They found that the response speed can be regulated by adjusting the surface/volume (*S/V*) ratio of PEDOT:PSS. The fastest response (<3.5 s) was achieved by using nanowires with a maximum S/V ratio. By doping PEDOT: PSS nanowires with graphene oxide (GO), its thermosensitivity can be maximized at a specific doping ratio. For the monitoring system based on wearable flexible temperature sensors to monitor temperature and other vital signals, secure attachment and strong biocompatibility are essential in obtaining accurate physiological and pathological information. Yamamoto et al., proposed a gel-free adhesive conductive electrode for electrocardiogram (ECG) recording and studied the influence of film thickness of the substrate on the temperature detection of the skin [96]. The resistive temperature sensor fabricated through a printing technique on a thin flexible film can monitor dehydration or heat stroke symptoms. Meanwhile, the gel-less system can effectively solve the problem of traditional gels that cannot be stably connected to the skin for a long time. Besides the adhesion problem, in order to obtain an accuracy results of skin temperature measurement, quantitative imaging of wound temperature with 2D/3D resolution is also very essential and important. Hattori et al. developed a conformal electronic platform based on thermal sensors and actuators that provide highly accurate and time-dependent mapping of temperature near wounds [97]. This type of wearable platform has provided a new way of fabricating biocompatible and noninvasive smart dressing for continuous monitoring of wounds.

By adding additional flexible wearable sensors or/and actuators to measure other biochemical health markers in/out the body and deliver the drug with precise control, the potential applications of the wearable sensors can now be widely developed and realized for human healthcare. In addition to monitoring human skin temperature, research shows new functions of wearable smart wound dressing. Kim et al., described a system containing two ultrathin and bendable temperature sensors and microscale heating elements used for medical sutures [98]. One suture line based on polyester fabric strip coated with PDMS contains silicon diode temperature sensors and Au micro-heaters. The other suture line contains 8 points Pt resistor temperature sensors formed between Au electrodes. This strategy enables a new class of wound sutures with flexible electronics that can monitor the state of the wound and provide therapeutic operations. If the wounds are severely infected and not treated in time, more serious damage and loss may occur. Along with real-time monitoring of wound status, rapid and effective treatment are also vitally and urgently needed to reduce any aggravation due to wound infection. To achieve a timely therapeutic intervention, different triggerable drug delivery systems were integrated with wearable sensors to form multifunctional smart wound dressing for both monitoring and treatment. Gong et al., prepared a moxifloxacin hydrochloride (MOX)-loaded polymer nanomesh film through an electrospinning method, and the thermoresponsive polymer nanomesh film had excellent flexibility and breathability (Figure 6b) [99]. This wearable temperature sensor can monitor the wound status in real-time by elevating temperature as an indicator of infection or inflammation at the wound site. Furthermore, the polymer nanomesh assembled with an efficient wearable heater that can be triggered to an on-demand release of the MOX loaded in the nanofibers for the timely therapeutics. Besides the thermal triggered drug release approach, Honda et al., proposed a drug delivery system based on a drug delivery pump made by a soft lithography technique connected with a microchannel for drug discharge [100]. In a recent study, Pang et al., have reported a flexible wearable electronics-integrated wound dressing, which consists of a temperature sensor, ultraviolet (UV) light-emitting diodes and a UV-responsive antibacterial hydrogel, for wound diagnosis and on-demand treatment (Figure 6c) [101]. Based on animal experiments, the wound dressing could diagnose early infection through continuous wound-temperature monitoring by the integrated temperature sensor, and provide timely treatment by releasing antibiotics from the hydrogel using in-situ UV irradiation (Figure 6d). This proof-of-concept smart dressing holds great promise in overcoming the “Black Box” of the wound-healing status and developing new approaches to significantly improve wound monitoring, diagnoses and treatments. Therefore, wound dressings with real-time monitoring and timely on-demand drug delivery capabilities are the most important directions in the development of wound rehabilitation treatment.

Besides the resistive temperature sensors, the wearable temperature sensors based on other mechanisms (e.g., capacitance, impendence, thermomagnetic) are also being widely studied. Recently, Lu et al., developed an LC (inductance-capacitance) oscillator that can measure regional body temperature through changes in capacitance [102]. The capacitor based on absorbable materials optimizes the access issues of some surgical implant devices, and effectively reduces the work of subsequent dressing removal after wound healing. Comparison of the sensitivity/accuracy of the abovementioned wearable temperature sensors based on different sensing mechanisms are summarized in Table 2. Taking into account the diversity and complexity of wound healing status and treatment, more smart diagnosis and pathological systems based on flexible, biocompatible and absorbable materials should be the focus in future research, and various types of smart wound dressing integrated with sensing components and drug delivery systems need further development to timely and effectively treat diseases.

## 4. Conclusions and Outlook

The development and implementation of wearable sensors and systems used for wound healing-related pH and temperature detection have been growing rapidly, and have begun to revolutionize the approach to wound diagnosis and treatment. Smart wound dressings integrated with wearable sensors to monitor the pH and temperature at the wound site in a noninvasive and real-time manner are promising in facilitating efficiencies in clinical resource use and management of distant patients. In addition, progresses in material science and manufacturing capacity have led to an endless emergence of new wound medical devices. Along with these advances, new types of wound dressing based on flexible, self-healable, absorbable materials and integrated with on-demand triggerable drug delivery systems will provide better and more efficient wound management and treatment.

Despite significant advances in recent years, the field of smart wound dressing integrated with wearable sensors and systems, which is really helpful for wound biomarkers (e.g., pH, temperature, etc.) monitoring and patient management, still needs to solve many bottlenecks to meet the requirements of modern healthcare. Due to subtle changes in wound pH and temperature when early infection occurs, the detection sensitivity of wearable sensors and the accuracy of the infection identification threshold require further improvement. Therefore, the next generation of medical wound dressings should focus on the improvement of sensitivity by applying novel materials or/and sensor structures, and the improvement of accuracy of wound infection judgment by integrating more biosensors to monitor multiple wound-related biomarkers apart from pH and temperature, providing mechanistic insights about the wound healing process and reliable basis for on-demand drug delivery. Moreover, the wound is a 3D structure, so 3D wound information is critical in the precision diagnosis of early infections. Thus, it is necessary to pay more attention to the development of large scale of pH or/and temperature sensors, which are fabricated through novel techniques and new protocols. Finally, wound rehabilitation is a long-term process in chronic wounds, which can always lead to the possibility of infection in the wound site. However, the wearable sensors mentioned above were tested in vitro or on human skin for only very short periods of time. Therefore, the long-term stability and reliability of the wearable sensors for wound monitoring are other important factors in ensuring long-term accurate detection. To overcome this obstacle, wearable sensors and systems with self-healing and self-adjustment properties need to be developed.

Overall, wearable sensors and systems have shown great potential in monitoring wound healing status through wound-related pH and/or temperature detection, which can effectively diagnose early infections and give timely on-demand therapeutics. Emerging solutions for each of the above limitations are also creating new opportunities for the advancement of wound dressings, and with the joint efforts made by researchers and doctors worldwide, the next generation of wound dressings based on wearable sensors and systems will provide more accuracy and reliable wound status monitoring and treatment.

## Figures and Tables

**Table 1:** Comparison of different wearable pH sensors based on different sensing techniques.

Analytical Technique	Sensing Materials	Range	Sensitivity/Accuracy
Optical-Fluorescence	5(6)-Carboxynaphthofluorescein	pH of 6–8 [34]	Intensity of a factor of ~40 between pH 6.0 and 7.7
Optical-Colorimetry	Curcumin	pH of 6–9 [36]	Actual pH was almost the same as calculated value
Electrochemical-Potentiometry	Polyaniline	pH of 4–10 [63]	−50 mV/pH
Electrochemical-Potentiometry	Graphite powder	pH of 6–9 [45]	4 mV/pH
Electrochemical-Impedance	CuO nanorod	pH of 5–8.5 [62]	0.64 μF/pH

**Table 2:** Comparison of different wearable temperature sensors based on different sensing techniques.

Analytical Technique	Sensing Materials	Range	Sensitivity/Accuracy
Optical-Thermochromic	Chiral nematic liquid crystal	26–40 °C [85]	~0.1 °C (accuracy)
Electrical-Resistive	Graphene	20–50 °C [89]	2.1% °C^−1^ (sensitivity)
Electrical-Resistive	Mxene	25–50 °C [18]	0.09% °C^−1^ (sensitivity)
Electrical-Capacitance	Polyethylene glycol	34–42 °C [102]	~0.5 °C (accuracy)
